# Role of the cholinergic system in the psychopathology and treatment of schizophrenia: a protocol for a scoping review

**DOI:** 10.3389/fpsyt.2025.1593211

**Published:** 2025-06-03

**Authors:** Michal Haczkiewicz, Ulrika Hylén, Mussie Msghina

**Affiliations:** ^1^ School of Medical Sciences, Örebro University, Örebro, Sweden; ^2^ Unit for Clinical Studies, Region Örebro County University Hospital, Örebro, Sweden; ^3^ Department of Psychiatry, Faculty of Medicine and Health, Örebro University, Örebro, Sweden; ^4^ Department of Clinical Neuroscience, Karolinska Institute, Stockholm, Sweden

**Keywords:** acetylcholine, cholinergic, cognition, emotion, motivation, schizophrenia, scoping review

## Abstract

**Introduction and objective:**

The cholinergic system has broad implications for affective and cognitive processes, which makes it pertinent for the psychopathology and treatment of mental disorders. Questions concerning its role in schizophrenia, a chronic disorder characterized by psychosis, emotional blunting and cognitive deficits, have been made particularly relevant due to the recent Food and Drug Administration (FDA) approval of a muscarinic agonist as an antipsychotic agent. The present paper details the protocol for a scoping review that will map models, evidence and research gaps concerning the role of the cholinergic system in the positive, negative and cognitive domains implicated in the psychopathology of schizophrenia.

**Methods and analysis:**

The scoping review will be conducted according to JBI (formerly the Joanna Briggs institute) methodology, using articles from the following databases: PubMed, Embase, Scopus, Cochrane Central Register of Controlled Trials and PsycInfo. Two independent reviewers will screen the articles using title and abstract, after which full text-analysis will determine inclusion. Only published original peer-reviewed English-language studies from the last 20 years that pertain to the review objective will be included. Clinical studies will be assessed for methodological quality and risk of bias. The results, which the reviewers will extract independently of each other using a data extraction tool, will be presented in accordance with the Preferred Reporting Items for Systematic Reviews and Meta-analysis Protocol Extension for Scoping Reviews (PRISMA-ScR).

**Discussion:**

Clarifying the research gaps in the field can indicate where future pre-clinical and clinical studies and systematic reviews can be worthwhile, and the risk of bias assessment aids in this by stratifying the included clinical trials according to quality. However, the language and publication date restrictions risk excluding relevant studies, which can introduce bias.

## Introduction

Schizophrenia is a chronic multifactorial developmental disorder whose psychopathology is characterized by distortions of reality and delusions (positive symptoms), reduction of emotional expression and amotivation (negative symptoms) and deficits in cognitive function (1). First- and second-generation dopamine D2-antagonists or partial agonists are the mainstay of treatment, and have been found to be more effective in treating the positive symptoms than the negative symptoms and cognitive deficits (2). The disorder typically debuts during young adulthood (3) and carries significant excess mortality (4) as well as disability, which, as quantified by Global Health Estimates 2019 (5) puts acute phase schizophrenia higher than an untreated spinal cord lesion at neck and severe multiple sclerosis.

The affected functional domains raise interest in the roles that the glutamatergic, serotonergic and cholinergic systems may have in the pathogenesis, pathophysiology and treatment of the disorder. The main object of the scoping review will be the significance of the central cholinergic system, which – in contrast with the relatively well-explored peripheral cholinergic signaling – remains to be elucidated. It has been hitherto implicated in a broad range of domains, including reward, attention, memory, cue detection (6), and the sleep-wakefulness cycle (7). This functional diversity is reflected in its anatomic localization, with cholinergic neurons originating in the basal forebrain spreading out diffusely throughout the neocortex (8) as well as localized cholinergic interneurons projecting to large swaths of the striatum (9), for instance.

Investigating the implications of cholinergic signaling has especially been made relevant in light of the Food and drug administration (FDA)-approval of Cobenfy for schizophrenia, which consists of the muscarinic M1 and M4 agonist xanomeline combined with the peripherally active muscarinic antagonist trospium (10). This represents a paradigm shift, as all antipsychotic treatment until now has consisted of dopamine D2-receptor antagonism, with the possible exception of the 5-hydroxytryptamine 2A receptor antagonist pimavanserin for Parkinson’s disease-related psychosis (11). Clinical experience has also contributed with acetylcholine-related clinical phenomena in schizophrenic patients, including patients on antipsychotic medication having a tendency to abuse anticholinergics (12), the curious pharmacology of the clozapine metabolite N-desmethylclozapine, a muscarinic agonist (13), and the lack of evidence for its antipsychotic effects in monotherapy (14), and the similarities between the effects of anticholinergic deliriants and the positive symptoms of schizophrenia.

Considering the significant burden of the disorder, the emerging pharmacological therapy options and the aforementioned clinical phenomena, it is pertinent for our planned scoping review to map the main models, concepts, evidence and gaps in the literature concerning the role of the neurotransmitter acetylcholine in the domains implicated in the positive, negative and cognitive symptoms of schizophrenia (hereafter referred to as the positive, negative and cognitive domains). This *a priori* protocol will ensure that the review is conducted transparently and that the authors can be held accountable by serving as a point of reference against which any modifications must be contrasted and by allowing the methodology to be scrutinized before it is implemented.

A PubMed search was conducted on the 18^th^ of February 2025 in order to identify already existing scoping reviews on the topic, with zero results.

## Methods and analysis

This protocol was developed using the JBI Evidence Synthesis Best Practice Guidance and Reporting Items for the Development of Scoping Review Protocols (15). The scoping review will be conducted according to the JBI Manual for Evidence Synthesis (16) and reported using the Preferred Reporting Items for Systematic Reviews and Meta-Analyses extension for Scoping Reviews (PRISMA-ScR) (17).

### Objectives

The aim of this scoping review will be to map the models, evidence and research gaps concerning the role of the cholinergic system in the affective and cognitive processes implicated in schizophrenia to facilitate future research questions.

The specific objectives can be summarized as follows:

1. Identify and map the available evidence concerning the role of the cholinergic system in the positive, negative and cognitive domains implicated in schizophrenia.
2. Map the quality of the included clinical studies by performing an assessment of their respective methodologies and risk of bias.
3. Identify and clarify the models and concepts that exist in the literature concerning this topic.
4. Identify the gaps in the existing research on this topic.

Applying the population, concept and context (PCC) formula recommended by JBI yields the following:

- Population: Only applicable to the clinical studies, which will include patients clinically diagnosed with schizophrenia according to the Diagnostic and Statistical Manual of Mental Disorders, Fourth Edition, Text Revision or Fifth Edition and/or International Classification of Diseases and Related Health Problems, Tenth or Eleventh Revision.
- Concept: The focus of this scoping review will be to map the models, evidence and research gaps concerning the role of the central nervous system (CNS) cholinergic system in the positive, negative and cognitive domains implicated in schizophrenia.
- Context: Peer reviewed published pre-clinical and clinical research from the last 20 years in English that pertains to the concept of this review.

### Eligibility criteria

Published pre-clinical and clinical peer reviewed original studies in English that examine the role of the cholinergic system in the CNS as it pertains to the positive, negative and/or cognitive domains implicated in schizophrenia will be considered for inclusion.

Given the recent years’ rapid development of techniques such as optogenetics, positron emission tomography–computed tomography, and functional magnetic resonance imaging, a publication date restriction that excludes studies older than 20 years will be applied.

Due to the explorative nature of the review, modifications of the eligibility criteria may be required to broaden or narrow the scope of the search. If applicable, they will be published in an appendix.

### Literature search

The literature search involves two stages: The initial stage 1 search in PubMed identified search terms for the stage 2 search, detailed below, by extracting relevant concepts, interventions and methodologies from the titles and abstracts of the included studies.

Stage 1 search terms included: Cholinergic, Pro-cholinergic, Acetylcholine, Muscarinic agonist, Nicotinic agonist, Anticholinergic, Anticholinergic withdrawal symptoms, Muscarinic antagonist, Nicotinic antagonist, Cognition, Psychosis, Delusion, Positive symptoms, Negative symptoms, Reward, Side-effect, Working memory, Attention, Emotion. The full stage 1 search, along with the extracted terms for the stage 2 search, are included in **Supplementary Material S1**, which is attached to the protocol.

### Selection of sources

The stage 2 search will be conducted in PubMed, Embase, Scopus, Cochrane Central Register of Controlled Trials (CENTRAL) and PsycInfo, after consultations with the Örebro University Medical Library librarian. The PubMed search, which is published in **Supplementary Material S2**, includes title and abstract terms and equivalent Medical Subject Headings (MeSH) terms from the stage 1 search which will be adapted to fit the terminology and syntax of the other search engines, for instance the American Psychological Association Thesaurus of Psychological Index Terms, Emtree terms or the CENTRAL truncation syntax. The non-PubMed searches may be modified to limit the number of irrelevant articles, which will be documented in an appendix.

Two independent reviewers will screen the articles for relevance according to the eligibility criteria using title and abstract, after which reading the full text will determine whether the article will be included or not. Discrepancies in screening and/or inclusion will be addressed through discussion between the reviewers. If the discrepancy cannot be resolved, the article will be carried to the full text-examination and assessed for inclusion. Further discrepancy beyond this point will be resolved by a third reviewer.

A screening pilot will be performed using 20 articles, after which the process will be evaluated to determine whether the eligibility criteria need to be modified or clarified. Modifications may also be made during the search upon identification of additional relevant search terms. Any such modifications will be published in an appendix.

Covidence (Veritas Health Innovation, Melbourne, Australia) will be used by both reviewers to manage the process of screening, evaluating and selecting the study material. Zotero (Corporation for Digital Scholarship, Vienna, Virginia) will be used to store and manage the references and to compile the reference list in the manuscript.

### Data extraction

The data extraction tool, as detailed in **Table 1**, will be piloted on five included studies to assess congruence between the independent reviewers and make modifications and/or clarifications, which will be detailed in a separate appendix if applicable. Due to the explorative nature of this scoping review, modifications may also be made to include categories of information that were not considered relevant at the outset, but that become pertinent during the data extraction. Any such modifications will be published in an appendix.

**Table 1:** The data extraction tool that will be used for the included studies.

Data item	Comments
Author(s) and date of acceptance	
Title of study	
Publication	
Country/region	
Study design	
Study population	If applicable
Postulated theoretical models relevant for role of cholinergic system in the positive, negative and/or cognitive domains implicated in schizophrenia	If applicable
Concepts relevant for the topic	If applicable
Identified gaps in research on the topic	If applicable
Evidence on the topic	
Primary and secondary outcomes of clinical studies on the topic	If applicable
Risk of bias assessment	If applicable
Risk of bias assessment tool used	If applicable

For clinical trials, more data may be required to perform a quality assessment according to the corresponding Swedish Agency for Health Technology Assessment and Assessment of Social Services risk-of-bias tool.

### Data presentation and analysis

A summary of the included studies, including their characteristics, will be presented in table form, and a PRISMA flow diagram will be used to visualize the screening and inclusion process. Figures may be used to visualize the identified models and concepts. The extracted data will be presented descriptively according to the relevant pharmacodynamic mechanisms of action, anatomical structures and functional – that is, positive, negative and cognitive – domains. The sources that are excluded at the full-text analysis stage will be presented in an appendix with reasons for exclusion.

The quality assessment of included clinical studies will be performed using the corresponding assessment tool from the Swedish Agency for Health Technology Assessment and Assessment of Social Services (SBU) (18) and reported descriptively in the results section. The heterogeneity of these studies in sample size and population and potential biases stemming from the methodology – as well as how they could impact any conclusions drawn – will be addressed in the discussion section. It is often noted, for example, that recruiting patients with psychotic disorders and preventing attrition among them is challenging, which risks introducing selection and survivorship biases. Given that enough comparable clinical trials are included, a meta-analysis of relevant primary and secondary outcomes and side-effect profiles will be performed.

## Discussion

This protocol details a scoping review that will seek to comprehensively map the role of the cholinergic system in the domains implicated in the positive, negative and cognitive symptoms of schizophrenia. The protocol details measures taken to mitigate some potential practical issues in conducting the review, including pilots for the screening and inclusion processes, a data extraction tool, and use of reference management software. Notwithstanding, the explorative nature of scoping reviews necessarily introduces uncertainty and subjectivity concerning, for instance, what qualifies as a “relevant concept” or when exactly the review should be broadened upon encountering pertinent information. The choices made in these situations will be detailed in an appendix. In addition, the wide range of clinical articles that will be included presents a challenge in adequately and fairly assessing the risks of bias, which may necessitate that the authors seek assistance of experts in the relevant subject matter.
