# Supplementary material for: Role of the cholinergic system in the psychopathology and treatment of schizophrenia: a protocol for a scoping review
Source: Front Psychiatry. 2025 Jun 3;16:1593211. doi: 10.3389/fpsyt.2025.1593211 (PMC12172546; doi:10.3389/fpsyt.2025.1593211)
Supplement: Supplementary file 1 [file DataSheet1.docx]

Supplementary material S1: Stage 1 search

Method:

A first PubMed search was performed on the 15^th^ of March, which was complemented with a second search on the 17^th^ of March. The first search required the articles to have relevant title and abstract terms *and* Medical Subject Headings (MeSH) terms to identify a limited set of articles with a high chance of relevance. This was deemed inadequate, however, due to too few included terms pertaining to positive and negative domains, which motivated a second search, this time using only title and abstract terms and limiting the scope to the last 20 years.

The terms were extracted from the titles and abstracts of the included articles according to whether they were deemed relevant by the corresponding author to the objectives of the review.

Search string for first stage 1 search

("Cholinergic"[Title/Abstract] OR "Pro-cholinergic"[Title/Abstract] OR "Acetylcholine"[Title/Abstract] OR "muscarinic agonist"[Title/Abstract] OR "nicotinic agonist"[Title/Abstract] OR "Anticholinergic"[Title/Abstract] OR ((("Cholinergic"[All Fields] AND "antagonists"[All Fields]) OR "cholinergic antagonists"[All Fields] OR "Anticholinergic"[All Fields] OR "anticholinergics"[All Fields]) AND "withdrawal symptoms"[Title/Abstract]) OR "muscarinic antagonist"[Title/Abstract] OR "nicotinic antagonist"[Title/Abstract]) AND (("Psychosis"[Title/Abstract] OR "Psychotic"[Title/Abstract]) AND ("Cognition"[Title/Abstract] OR "Delusion"[Title/Abstract] OR "positive symptoms"[Title/Abstract] OR "negative symptoms"[Title/Abstract] OR "Reward"[Title/Abstract] OR "side effect*"[Title/Abstract] OR "working memory"[Title/Abstract] OR "Attention"[Title/Abstract] OR "Emotion"[Title/Abstract])) AND ("Cholinergic Neurons"[MeSH Terms] OR "Cholinergic Agents"[MeSH Terms] OR "Cholinergic Antagonists"[MeSH Terms] OR "Cholinergic Agonists"[MeSH Terms] OR "Cholinergic Fibers"[MeSH Terms] OR "Muscarinic Agonists"[MeSH Terms] OR "Muscarinic Antagonists"[MeSH Terms] OR "Nicotinic Agonists"[MeSH Terms]) AND ("Psychotic Disorders"[MeSH Terms:noexp] OR "Cognitive Dysfunction"[MeSH Terms:noexp] OR "Cognition"[MeSH Terms] OR "Delusions"[MeSH Terms] OR "Attention"[MeSH Terms] OR "memory, short term"[MeSH Terms] OR "Executive Function"[MeSH Terms])

Date of PubMed search: 15^th^ of March 2025. 39 studies were included for screening, of which 7 were deemed irrelevant and were excluded from the extraction.

Extracted terms from first stage 1 search

Table 1: The terms extracted from the titles and abstracts included in the first stage 1 PubMed search and the number of articles in which the terms occurred.

| **Term** | **Number of articles in which the term occurred, or (comment)** |
| --- | --- |
| Cognitive OR Neurocognitive | 28 |
| Antipsychotic | 19 |
| “Anticholinergic burden” OR “Anticholinergic medication burden” OR “Anticholinergic load” | 12 |
| “Verbal memory” | 5 |
| “Processing speed” OR “Speed of information processing” | 4 |
| Attentional | 4 |
| Xanomeline OR “Xanomeline-trospium” | 3 |
| “Brief Assessment of Cognition in Schizophrenia” | 3 |
| “Anticholinergic Burden Scale” | 2 |
| “Visual learning” | 2 |
| Scopolamine | 2 |
| “Latent inhibition” | 2 |
| “Anticholinergic Drug Scale” | 2 |
| “Prepulse inhibition” | 2 |
| “Gating deficit*” | 2 |
| “Basal forebrain” OR “Basal forebrain cholinergic nuclei” | 2 |
| Neuroleptic* OR “Neuroleptic drug*” | 1 |
| “Reaction time” | 1 |
| F20 (schizophrenia diagnosis) | 1 |
| “Antimuscarinic syndrome” | 1 |
| “Attentional perseveration” | 1 |
| Deliri* | 1 |
| “Social cognition” | 1 |
| Endophenotype | 1 |
| “Muscarinic cholinergic hypothesis theory” | 1 |
| “Episodic memory” OR “Delayed episodic memory” | 1 |
| Antihistamine* | 1 |
| “Anticholinergic Risk Scale” | 1 |
| Varenicline | 1 |
| Antisaccade | 1 |
| P50 OR “Sensory gating” OR “P50 Sensory gating” | 1 |
| “Sustained attention” | 1 |
| Emraclidine | 1 |
| “Antimuscarinic psychosis” | 1 |
| Propsychotic | 1 |
| “Functional connectivity” | 1 |
| Pedunculopontine | 1 |
| “Laterodorsal tegment*” | 1 |
| “Smooth pursuit” | (Extrapolated from “predictive pursuit” and “maintenance pursuit”) |

Search string for second stage 1 search

("Cholinergic"[Title/Abstract] OR "Pro-cholinergic"[Title/Abstract] OR "Acetylcholine"[Title/Abstract] OR "muscarinic agonist*"[Title/Abstract] OR "nicotinic agonist*"[Title/Abstract] OR "Anticholinergic"[Title/Abstract] OR "muscarinic antagonist"[Title/Abstract] OR "nicotinic antagonist"[Title/Abstract]) AND ("positive symptoms"[Title/Abstract] OR "negative symptoms"[Title/Abstract]) Filters: Clinical Trial, Randomized Controlled Trial, Review, Systematic Review, from 2005 – 2025

Date of PubMed search: 17^th^ of March. 90 studies were included for screening, of which 25 were deemed irrelevant and were excluded from the extraction.

Extracted terms from second stage 1 search

Table 2: The terms extracted from the titles and abstracts included in the second stage 1 PubMed search and the number of articles in which the terms occurred.

| **Term** | **Number of articles in which the term occurred** |
| --- | --- |
| “Positive allosteric modulator” | 11 |
| Nicotine | 10 |
| Hallucinations | 6 |
| Anhedonia | 5 |
| “Positive and Negative Syndrome Scale” | 4 |
| “Scale for the Assessment of Negative Symptoms” | 4 |
| Avolition OR Apathy | 4 |
| Alogia OR Aprosody | 4 |
| “alpha7 NNR” OR “Alpha(7)-nicotinic receptor” OR “α7 nicotinic acetylcholine receptors” OR “α7-nicotinic acetylcholine receptor” | 4 |
| Galantamine | 3 |
| “Consensus Cognitive Battery” | 3 |
| Donepezil | 3 |
| “Alpha-7 nicotinic receptor agonist*” OR “α7 nicotinic receptor agonist*” OR “α7-nicotinic receptor positive allosteric modulator*” | 3 |
| Amotivation | 2 |
| “Mismatch negativity” | 2 |
| “Brief Psychiatric Rating Scale” | 2 |
| “Self-medication hypothesis” | 1 |
| “Novelty detection” | 1 |
| “Measurement and Treatment Research to Improve Cognition in Schizophrenia” | 1 |
| “Digital Symbol Substitution test” | 1 |
| “Stroop test” | 1 |
| “Continuous Performance Test” | 1 |
| “Wisconsin Card Sorting Test” | 1 |
| “Emotional expression” | 1 |
| Asociality | 1 |
| “Subtype-specific allosteric modulators” | 1 |
| “Response inhibition” | 1 |
| “Sensorimotor gating” | 1 |
| “Nicotinic-glutamatergic interactions” | 1 |
| “Hypoglutamatergic hypothesis” | 1 |
| “Cortical acetylcholine depletion” OR “Cortical ACh depletion” OR “Cortical acetylcholine (ACh) depletion” | 1 |
| “Monoaminergic-muscarinic imbalance” | 1 |
| “Cognitive impairments associated with schizophrenia” | 1 |
| “Quintuple hypotheses” OR “dopamine, nicotinic-cholinergic, glutamatergic/NMDA, GABA, and KYNA” | 1 |
| “Muscarinic blockade” | 1 |
| “Striatal cholinergic interneurons” | 1 |
| “Repeatable Battery for the Assessment of Neuropsychological Status” | 1 |
| “Novel object recognition” | 1 |
| “Cholinergic receptor modulator” | 1 |
| “Clinical Antipsychotic Trials of Intervention Effectiveness” | 1 |
| “Islands of Calleja” | 1 |
| “Reality distortion” | 1 |
| “Thought disorders” | 1 |
| “Corticolimbic circuit” OR “Cortico-limbic circuit” | 1 |
| “N-methyl-d-aspartate (NMDA) receptor hypofunction hypothesis” | 1 |
