# Supplementary material for: Role of the cholinergic system in the psychopathology and treatment of schizophrenia: a protocol for a scoping review
Source: Front Psychiatry. 2025 Jun 3;16:1593211. doi: 10.3389/fpsyt.2025.1593211 (PMC12172546; doi:10.3389/fpsyt.2025.1593211)
Supplement: Supplementary file 2 [file DataSheet2.docx]

Supplementary material S2: Stage 2 PubMed search

# Method

A PubMed search was conducted on the 26^th^ of March 2025, yielding 708 results. The search is composed of a cholinergic and a non-cholinergic block. Terms that were deemed too broad, that is, “cognition” (and synonyms), “endophenotype”, “attention” and “functional connectivity”, were included in an AND-block with “schizophrenia” to narrow the search. However, specific cognitive domains such as “working memory” or “response inhibition” were kept in the main non-cholinergic block.

Search string for PubMed stage 2 search

((("Cholinergic"[Title/Abstract] OR "Pro-cholinergic"[Title/Abstract] OR "Acetylcholine"[Title/Abstract] OR "muscarinic agonis*"[Title/Abstract] OR "nicotinic agonis*"[Title/Abstract] OR "Anticholinergic"[Title/Abstract] OR "muscarinic antagonis*"[Title/Abstract] OR "nicotinic antagonis*"[Title/Abstract] OR "muscarinic receptor*"[Title/Abstract] OR "nicotinic receptor*"[Title/Abstract] OR "Anticholinergic burden"[Title/Abstract] OR "Anticholinergic medication burden"[Title/Abstract] OR "Anticholinergic load"[Title/Abstract] OR "Antimuscarinic syndrome"[Title/Abstract] OR "Antimuscarinic psychosis"[Title/Abstract] OR "alpha7 NNR"[Title/Abstract] OR "alpha 7 nicotinic receptor"[Title/Abstract] OR "alpha7 nicotinic acetylcholine receptors"[Title/Abstract] OR "alpha7-nicotinic acetylcholine receptor"[Title/Abstract] OR "Muscarinic blockade"[Title/Abstract] OR "Cortical ACh depletion"[Title/Abstract] OR ("Cholinergic Agents"[MeSH Terms] OR "Acetylcholine"[MeSH Terms] OR "receptors, nicotinic"[MeSH Terms] OR "receptors, muscarinic"[MeSH Terms] OR "Anticholinergic Syndrome"[MeSH Terms] OR "Basal Forebrain"[MeSH Terms] OR "Pedunculopontine Tegmental Nucleus"[MeSH Terms])) AND ("Verbal memory"[Title/Abstract] OR "Processing speed"[Title/Abstract] OR "Speed of information processing"[Title/Abstract] OR "Visual learning"[Title/Abstract] OR "Reaction time"[Title/Abstract] OR "Social cognition"[Title/Abstract] OR "Episodic memory"[Title/Abstract] OR "Delayed episodic memory"[Title/Abstract] OR "Antisaccade"[Title/Abstract] OR "Sustained attention"[Title/Abstract] OR "Response inhibition"[Title/Abstract] OR "Smooth pursuit"[Title/Abstract] OR "Novelty detection"[Title/Abstract] OR "Mismatch negativity"[Title/Abstract] OR "Stroop test"[Title/Abstract] OR "Continuous Performance Task"[Title/Abstract] OR "Continuous Performance Test"[Title/Abstract] OR "Wisconsin Card Sorting Test"[Title/Abstract] OR "Novel object recognition test"[Title/Abstract] OR "Working memory"[Title/Abstract] OR ("positive symptom*"[Title/Abstract] OR "Latent inhibition"[Title/Abstract] OR "Antipsychotic"[Title/Abstract] OR "Propsychotic"[Title/Abstract] OR "Pro-psychotic"[Title/Abstract] OR "Hallucinations"[Title/Abstract] OR "Reality distortion"[Title/Abstract] OR "Psychosis"[Title/Abstract] OR "Delusion"[Title/Abstract]) OR ("Negative symptoms"[Title/Abstract] OR "Anhedonia"[Title/Abstract] OR "Avolition"[Title/Abstract] OR "Amotivation"[Title/Abstract] OR "Emotional expression"[Title/Abstract] OR "Asociality"[Title/Abstract]) OR ("Sensorimotor gating"[Title/Abstract] OR "Prepulse inhibition"[Title/Abstract] OR "gating deficit*"[Title/Abstract] OR "sensory gating"[Title/Abstract]) OR ("Brief Assessment of Cognition in Schizophrenia"[Title/Abstract] OR "Anticholinergic Burden Scale"[Title/Abstract] OR "Anticholinergic Drug Scale"[Title/Abstract] OR "Anticholinergic Risk Scale"[Title/Abstract] OR "Positive and Negative Syndrome Scale"[Title/Abstract] OR "Consensus Cognitive Battery"[Title/Abstract] OR "Brief Psychiatric Rating Scale"[Title/Abstract] OR "Measurement and Treatment Research to Improve Cognition in Schizophrenia"[Title/Abstract] OR "Repeatable Battery for the Assessment of Neuropsychological Status"[Title/Abstract] OR "Clinical Antipsychotic Trials of Intervention Effectiveness"[Title/Abstract]) OR "deliri*"[Title/Abstract] OR "thought disorder*"[Title/Abstract] OR "disordered thought*"[Title/Abstract] OR (("Cognitive"[Title/Abstract] OR "Cognition"[Title/Abstract] OR "Neurocognitive"[Title/Abstract] OR "Neurocognition"[Title/Abstract]) AND "schizophreni*"[Title/Abstract]) OR (("endophenotyp*"[Title/Abstract] OR "attenti*"[Title/Abstract] OR "Functional connectivity"[Title/Abstract]) AND "schizophreni*"[Title/Abstract]) OR (("memory, short term"[MeSH Terms] OR "memory, episodic"[MeSH Terms] OR "Anhedonia"[MeSH Terms] OR "pursuit, smooth"[MeSH Terms] OR "Hallucinations"[MeSH Terms:noexp] OR "Delusions"[MeSH Terms] OR "Apathy"[MeSH Terms] OR "Delirium"[MeSH Terms:noexp] OR "sensory gating"[MeSH Terms] OR "Auditory Perceptual Disorders"[MeSH Terms] OR ("Cognition"[MeSH Terms] OR "Endophenotypes"[MeSH Terms])) AND "Schizophrenia Spectrum and Other Psychotic Disorders"[MeSH Terms]))) NOT ("Review"[Publication Type] OR "systematic review"[Publication Type] OR "scoping review"[Publication Type])) AND (("all"[Filter] NOT "preprint"[Publication Type]) AND ("clinical study"[Publication Type] OR "clinical trial"[Publication Type] OR "comparative study"[Publication Type] OR "controlled clinical trial"[Publication Type] OR "randomized controlled trial"[Publication Type]) AND "english"[Language] AND 2005/01/01:2025/12/31[Date - Publication])
